# Social Network Position Moderates the Relationship between Late-life Depressive Symptoms and Memory Differently in Men and Women

**DOI:** 10.1038/s41598-019-42388-3

**Published:** 2019-04-16

**Authors:** Hairin Kim, Seyul Kwak, Junsol Kim, Yoosik Youm, Jeanyung Chey

**Affiliations:** 1 Department of Psychology, Seoul National University, Seoul, South Korea; 2 Department of Sociology, Yonsei University, Seoul, South Korea

## Abstract

Late-life depression has been considered to be associated with memory deficits and hippocampal volume reduction. Considering that not all depression patients undergo the same amount of cognitive impairment or regional brain volume loss, moderating factors such as complex mental activity and social activity have been examined to assess whether these factors attenuate the detrimental impact of depressive symptoms on cognitive function and regional brain volume. However, the premise that a cognitively stimulating experience may modify the association between depressive symptoms and memory or hippocampal volume has not been investigated using social network data, which would reflect individuals’ concrete characteristic of everyday social activity. In a social network, a brokerage position which connects two otherwise unconnected others demands mental and physical efforts. Using complete social network data in an entire village in South Korea, we examined whether opportunities for brokerage in social networks alter the negative association between depressive symptoms and episodic memory function or hippocampal volume in older adults. Initially, 125 participants were included in the analysis involving episodic memory function. Then, of which 65 participants completed the MRI scan, and were included in the subsequent analysis containing the hippocampal volume. Furthermore, we investigated the gender-specific effect of brokerage based on the previously reported gender difference in the effect of social networks. We found a gender-specific moderating effect of brokerage. For men, a large opportunity for brokerage weakened the negative association between depressive symptoms and memory performance and left hippocampal volume. In contrast, women showed that a large opportunity for brokerage was not beneficial for assuaging the impact of depressive symptoms on memory performance and hippocampal volume. In women, the opportunity for brokerage was positively associated with the detrimental impact of depressive symptoms on memory performance and hippocampal volume. Our findings suggest that occupying a bridging position in a social network may minimize the impact of depressive symptoms on memory function and hippocampal volume among older men, whereas the opposite holds true for older women.

## Introduction

Late-life depression, including depressive symptoms, has been associated with both poor cognitive function and regional brain volume loss, particularly in the hippocampus^1–5^. In late-life depression, the memory loss generally occurs with hippocampal volume reduction and it is possibly mediated by hippocampal volume loss^6–9^. Also, depressive symptoms have predicted subsequent cognitive decline independently of the baseline cognitive impairment, implicating the detrimental influences of depressive symptoms^10^. However, the severity of memory impairment and degree of hippocampal volume atrophy varied across depression patients. Even among those diagnosed with depression, not all patients undergo the same amount of cognitive impairment and regional brain volume changes, including hippocampus^11,12^. The inconsistency of the severity of memory decline and hippocampal atrophy in depression indicates the possibility of the moderating factors in the relationship between depressive symptoms and its detrimental effects on memory function or hippocampal volume.

Although the inconsistency could be derived from the use of different depression scales or symptom severity, the existing bodies of work have emphasized the possible protective effect of a stimulating life-time experience in the late-life depression^13–15^. For instance, a meta-analysis has suggested that participating in a social activity or several types of complex mental activity may alleviate the negative influences of depressive mood on cognitive function among older adults^16–19^. Simultaneously, individual differences in the impact of depressive symptoms could be related to the buffering effect of social support. In the context of the buffering hypothesis of social support^20^, positive emotions perceived through social interactions may form a key role in cognitive function in aging^21^. However, socially isolated individuals are hard to buffer the impact of health stressors and consequently are at higher cognitive and mental health outcomes, while individuals with abundant social resources are able to buffer the stress^22,23^. The social support flows through an individual’s social network, hence the social network has been considered as a crucial element affecting health, including mental well-being^24^.

However, the studies that have investigated the role of social engagement, including social activity in late-life depression, have relied on self-reports. It has been suggested that assessing social activity through self-reports may be limited by subjective recall bias, particularly for individuals with cognitive difficulties such as older adults^25^. To deal with this problem, researchers have begun taking advantage of sociocentric methods, which allow research to quantify and uncover the patterns of structural social relationships and positions. Those characteristics, which are captured through sociocentric methods, are associated not only with social cognition and brain function^26–28^ but also with late-life health outcomes^29,30^.

**Figure 1:**
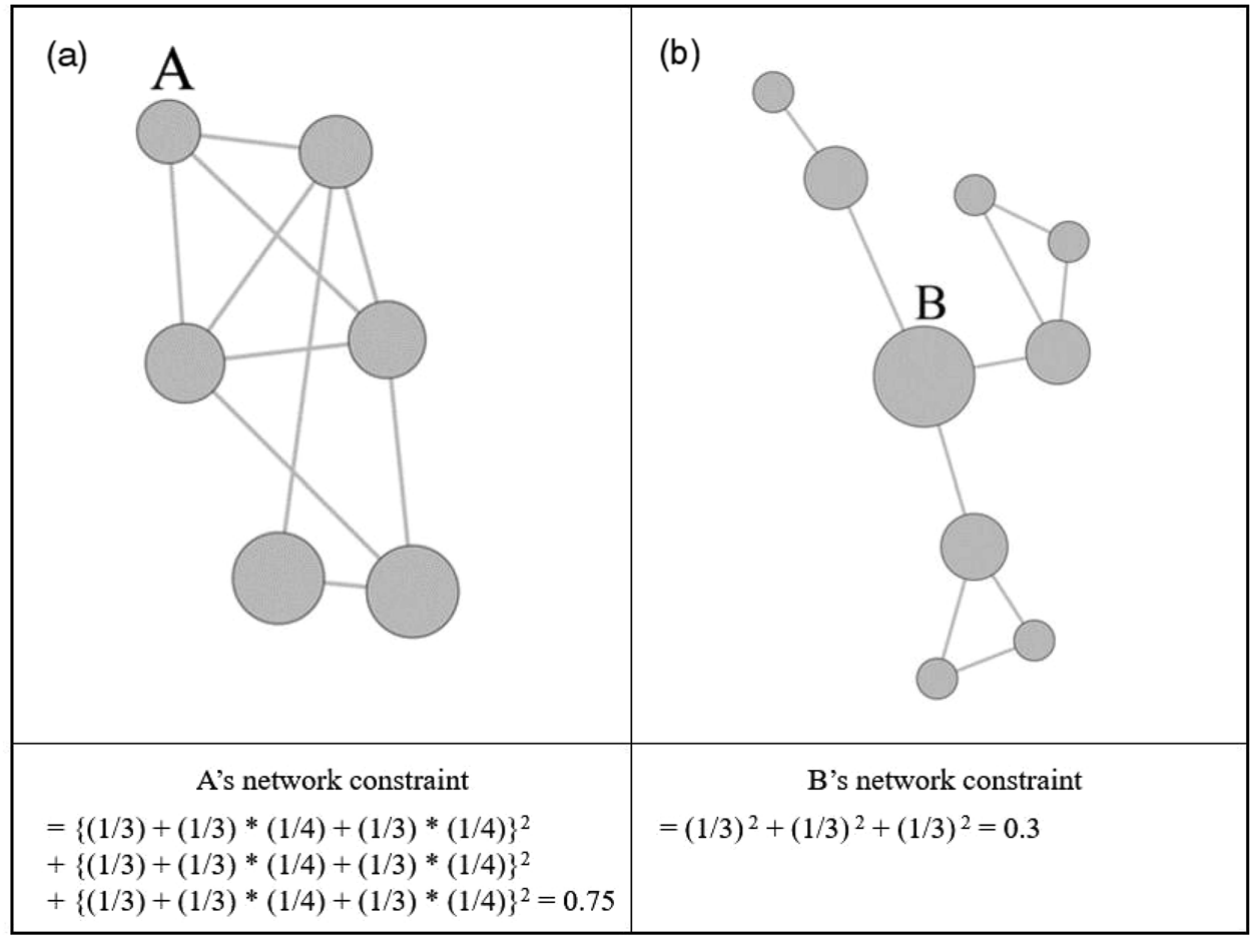
Example of a bridging position. B has a better opportunity for the brokerage role (**b**) than A (**a**). A larger circle size represents more opportunity for brokerage. By definition, brokerage opportunity refers to the smaller network constraint on an individual. The bottom panel shows the equation for each individual's social network constraint.

According to previous literature that highlights the role of complex mental activity in late-life depression, we focused on the bridging position involving complex mental activities in social network. Bridging position is often termed “brokerage” since it connects two otherwise unconnected others (Fig. 1). Similar to other complicated and demanding life experiences, brokerage demands greater physical and mental capacities since two parties who are not connected to each other have different and sometimes even conflicting social backgrounds^31,32^. For instance, an individual engaged in relationships with different kinds of social groups is more likely to face various demands concerning his or her time and energy from each group to keep the social relationships alive^33^. Given the heterogeneity of groups, which are governed by different and even contradictory social norms or beliefs, a bridging position between two distinct social groups is required to switch between different cognitive frameworks of each social group^34,35^. Based on these perspectives, Cornwell and colleagues (2009a) suggested that older adults with more opportunities for bridging unconnected others have better cognitive and physical health, implicating that physical capacity and cognitive skills are persistently required to transmit resources or information to other groups.

Situating between different social groups may be costly and facing conflicting roles may bring negative outcomes. Depending on a preference for social network type or a social status in social network, the bridging positon can be a double-edged sword. Indeed, in a study of adolescents, it was found that women pay significant psychological costs to maintain their brokerage positions^36^. In line with this, the adverse outcomes of brokerage in women have been consistently observed across different age groups^36–38^. One reason for assuming that the benefits of brokerage position can vary across gender is that men and women feel social support in different types of social network. Social network studies revealed that women tend to feel social support from the small and dense social network. Meanwhile, men are likely to get benefits from extensive and coarse social network^39,40^. Based on this phenomenon, for men, a brokerage position in a social network can be a mentally stimulating experience as well as a source of social support. However, for women, the brokerage position is likely to be a position requiring more psychological resources while providing less full-filling social support.

In this study, through the bridging position in a social network, we examined the contingent relation between depressive symptoms and memory function or hippocampal volume. Based on previous literature suggesting gender differences in social network, we hypothesized that the moderating role of bridging position could vary across genders. Specifically, we hypothesized that occupying brokerage positions would attenuate the harmful outcome of depressive symptoms only in men and possibly exacerbate its effect in women.

## Results

### Descriptive statistics

Table 1 shows the results of the correlation analysis of the main study variables and demographic variables, including age and years of education. Gender showed a significant correlation with years of education and intracranial volume. Depressive symptoms showed a negative correlation with both long-term memory (LTM) indices and left hippocampal volume. The LTM Recognition index showed a positive correlation with bilateral hippocampal volume. Individual’s social network size showed a negative correlation with the social network constraint measuring brokerage and it indicates that individuals with a large social network tend to have more opportunities for the brokerage position than the others. Skewness and kurtosis were within an acceptable range for all the dependent variables (range_skewness_ = −0.60 to 0.01; range_kurtosis_ = −0.54 to 0.69), indicating a satisfactory univariate normal distribution^41^.

**Table 1:** Correlation among demographic variables, depressive symptoms, brain volume, and social network.

	Variable	1	2	3	4	5	6	7	8	9	10
1	Gender^a^										
2	Age	0.09									
3	Education	0.52	−0.27**								
4	Depressive symptoms	−0.03	0.23**	−0.18*							
5	LTM Recall	−0.13	−0.33**	0.25**	−0.27**						
6	LTM Recognition	−0.04	−0.32**	0.36**	−0.37**	0.70**					
7	Brokerage	−0.11	−0.11	0.17	−0.05	0.08	0.17				
8	Social network size	0.10	0.03	−0.05	0.01	−0.09	−0.20	−0.63**			
9	Left hippocampus volume	0.14	−0.37**	0.26*	−0.29*	0.17	0.30*	0.12	−0.09		
10	Right hippocampus volume	0.08	−0.46**	0.22	−0.27*	0.21	0.35**	0.16	−0.11	0.79**	
11	Intracranial volume	0.54**	−0.12	0.52**	−0.27*	0.06	0.18	0.15	−0.02	0.56**	0.51**

Note. Correlation represent Pearson’s correlation except for gender; ^a^Spearman correlation coefficient represented for gender variable.

Depressive symptoms: Geriatric Depression Scale (GDS) total score; LTM: Long-Term Memory; Brokerage: Social network structural constraint (range 0–1), a smaller value indicates a larger opportunity for brokerage; Social network size: number of social ties in the global network.

**p < 0.001, *p < 0.05, 2-tailed.

### Neuropsychological test sample

Gender-specific regression models were generated to investigate whether the relation between depressive symptoms and memory performance is contingent on the brokerage measure among older adults. The analysis results are presented in Table 3. The two-way interaction term (depressive symptoms × brokerage) predicting LTM Recognition was significant among both older men (*p* = 0.04) and women (*p* = 0.01), indicating that the relation between depressive symptoms and memory performance was modified by brokerage role for both genders. The identical two-way interaction term in the model predicting the LTM Recall index was not significant for men or women.

In the subsequent analysis, the three-way interaction term (depressive symptoms × brokerage × gender) was significant in the model predicting the LTM Recognition index (*p* < 0.01). In an identical model predicting the LTM Recall index, the three-way interaction term was not statistically significant.

To test the robustness of the regression model predicting LTM Recognition, outlier detection was conducted using Cook’s distance. For men, after the removal of the data point of one participant who met the Cook’s distance outlier criteria, the statistical significance of the two-way interaction term was improved (*p* = 0.01). For women, no outlier was detected using the Cook’s distance criterion. Due to a solid correlation between social network size and the brokerage measure, the social network size covariate was registered as an additional covariate in the above models. The significance of the interaction terms was not undermined by accounting for the effect of social network size (men: B = −0.04, *p* = 0.04; women: B = 0.05, *p* < 0.01).

### Magnetic resonance imaging subsample

In the MRI subsample, a statistical analysis identical to that of the neuropsychological test sample was conducted to predict bilateral hippocampal volume. The analysis results are presented in Table 3. For men, the two-way interaction term (depressive symptoms × brokerage) predicting adjusted left hippocampal volume was marginally significant (*p* = 0.10). For women, this two-way interaction term was significant (*p* = 0.03). For both men and women, the two-way interaction term predicting adjusted right hippocampal volume was not statistically significant.

Subsequently, the three-way interaction term (depressive symptoms × brokerage × gender) was tested. In line with the analysis in the neuropsychological test sample, the three-way interaction term was significant (*p* = 0.01) in the model predicting adjusted left hippocampal volume. This result revealed that the moderation pattern differs significantly across gender.

**Figure 2:**
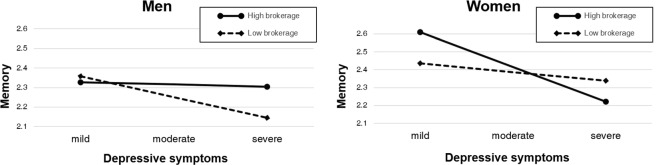
Moderation effect of brokerage in the association between depressive symptoms and memory function. Note. Long-Term Memory Recognition index as a function of depressive symptoms and brokerage (social structural constraint) in men (left) and women (right); Depressive symptoms score: mild (1 standard deviation below the mean in the sample population), moderate (mean in the sample population), moderate (mean in the sample population), severe (1 standard deviation above the mean in the sample population); High/Low brokerage was split for the purpose of visualization. High brokerage: 1 standard deviation above the mean in the sample population (constraint value = 0.26); Low brokerage: 1 standard deviation below the mean in the sample population (constraint value = 0.88).

**Figure 3:**
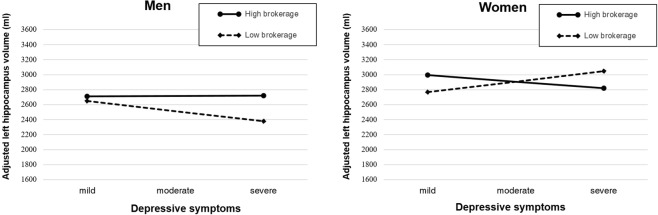
Moderation effect of brokerage in the association between depressive symptoms and adjusted left hippocampal volume. Note. Left hippocampal volume as a function of depressive symptoms and brokerage (social structural constraint) in men (left) and women (right); Depressive symptoms score: mild (1 standard deviation below the mean in the sample population), moderate (mean in the sample population), moderate (mean in the sample population), severe (1 standard deviation above the mean in the sample population); High/Low brokerage was split for the purpose of visualization. High brokerage: 1 standard deviation above the mean in the sample population (constraint value = 0.25); Low brokerage: 1 standard deviation below the mean in the sample population (constraint value = 0.86)

To test the robustness of the regression model, outlier detection was conducted using Cook’s distance. For men, two data points were classified as outliers based on Cook’s distance. After excluding two outlier data points, the two-way interaction term (depressive symptoms × brokerage) predicting adjusted left hippocampal volume was significant (*p* = 0.02). For women, one data point was detected as an outlier. After this data point was removed, the effect of two-way interaction was not significant. The significance of the interaction terms was not undermined by accounting for the effect of social network size (men: B = −41.98, *p* = 0.11; women: B = 59.31, *p* = 0.02). Figures 2 and 3 describe the moderation pattern of brokerage in men and women.

## Discussion

The present study is the first to examine the gender-specific role of bridging position in a social network in the context of late-life depression. Men with sufficient brokerage opportunities demonstrated an assuaged negative impact of depressive symptoms on memory performance and left hippocampal volume. In contrast, for women, more opportunities for brokerage were associated with greater detrimental impact of depressive symptoms on memory performance and hippocampal volume.

Given that a complex and cognitively stimulating life-time experience may play a role in attenuating the detrimental effects of late-life depressive symptoms on cognitive function and brain volume, we inferred that men who had more opportunity for brokerage in their social network were more likely to deal with and integrate diverse or even conflicting demands from their social ties and this opportunity for the brokerage position could weaken the negative association between depressive symptoms and episodic memory performance or hippocampal volume. In contrast, a buffering role of brokerage under the influence of depressive symptoms was not observed among women. Rather, women with more opportunity for brokerage showed greater adverse effects of depressive symptoms on their memory performance and hippocampal volume. Although the results found for women may not be consistent with the previous studies indicating advantages of bridging position^31,42^, the possibility of gender differences in brokerage has been discussed earlier because men tend to receive benefits from the brokerage position while women appear to be burdened by it^37^. The gender difference in brokerage could be attributed to the fact that socialization and affiliative relationship patterns differ between men and women^39,40^. Men tend to be satisfied with large and coarse social network in which they share their activities rather than building close emotional or supportive relationships. In contrast, women tend to rely on their social network for emotional, instrumental, and informational support^43^. Likewise, women are likely to receive more benefit from kin and other women rather than men, indicating that homophily may enhance the positive outcomes of social networks, such as physical and psychological health^39^. Consistent with the hypotheses, Carboni and Gilman^36^ found that brokerage was positively associated with social stress and negatively associated with life satisfaction among women. Therefore, in the women of the present study, it seems that the costs of brokerage may have far outweighed the advantages of bridging opportunity.

This study has examined the role of a social network position called “brokerage” in the context of late-life depression. Even though individuals cannot know their social network position, the social network position captures the unique characteristics of an individual’s daily social activity. By using a complete social network, we were able to define a social network position involving complex mental activity and found its benefits and costs in late-life depressive symptoms. Being located in a bridging position would require more energy and resources to keep complicated and conflicting relationships^33,34^. Therefore, men with brokerage seem to have benefited from their experience of a demanding social position when they have symptoms of depression. Nevertheless, we admit that we cannot eliminate the possibility that individuals who have better memory function or larger hippocampal volume are more likely to occupy the bridging position^44^ and that they would then show better memory performance or larger hippocampal volume under the influence of depression. However, we may still insist on our hypothesis, which is centered on the buffering role of brokerage, in that we could not find a simple correlation between the brokerage opportunity and memory performance or hippocampal volume in the data.

More broadly, our findings provide one plausible explanation for the individual difference in the severity of memory deficits and hippocampal atrophy under a similar severity of depressive symptoms. Given that depressed older adults with cognitive deficits are more likely to progress to dementia than those depressed older adults without severe cognitive deficits^45,46^, finding a moderating factor in the association among depressive symptoms, memory performance, and hippocampal volume is a meaningful achievement. Our results represent an important step forward in examining a moderating factor weakening the influence of depressive symptoms on cognitive impairment by adding a measurement of hippocampal volume. One of the possible neurobiological mechanisms of depression-related memory decline is the dysregulation of the hypothalamic-pituitary-adrenal axis and subsequent hippocampal volume loss^8,47^. However, cognitively stimulating life-time experience has been known to lessen age-related regional brain volume loss, including the hippocampus. In a longitudinal study, older adults who experienced complex mental activity during their lifetime showed slower hippocampal atrophy rate^48^. Moreover, in a cross-sectional study, time spent in cognitively engaging activities was associated with larger cortical and subcortical volume, including the hippocampus^49^. Therefore, the fact that older men with larger opportunity for brokerage showed reduced association between depressive symptoms and left hippocampal volume could be attributed to their cognitively stimulating experiences from demanding social position.

We note several limitations in this study. Firstly, the use of cross-sectional data limits the interpretation. The approach outlined in this study should be replicated in a longitudinal study to elucidate the pathway among depressive symptoms, memory, and hippocampal volume and the moderating influence of bridging position on this pathway. Even though it is possible that smaller baseline hippocampal volume may have contributed to the age-related cognitive dysfunction or psychopathologies^50^, animal studies supported the causal direction among stress, hippocampal atrophy, and cognitive decline^51^ by showing that aged rats exposed to chronic stress showed hippocampal dendritic atrophy and inhibited neurogenesis with memory decline. Secondly, even if we assessed the social network property of an entire village, the number of participants included in the analysis of neuropsychometry and regional brain volume was relatively small. In particular, the MRI subsample consisted of 65 older adults, with smaller number of men. Hence we cannot rule out the possibility that MRI analysis results may not be robust. Yet, the consistency of the results within each gender may minimize the possibility that the MRI subsample analysis result was unstable due to small sample size. Further, it may be inappropriate to generalize the findings to individuals with clinical depression since our results were based on self-reported late-life depressive symptoms in a community setting. Lastly, we found the moderation effect of brokerage only in the left hippocampus among the bilateral volumes. Although it may limit our finding, this is probably consistent with the previous finding that lateralization of hippocampal atrophy in patients with psychiatric disorders was related to the hypersecretion of glucocorticoids, including depression^52^.

In conclusion, social network characteristics, specifically brokerage position, measured using the entire population of a village, may play a protective role against the negative consequences of psychiatric disorders since there was a decreased impact of depressive symptoms on memory function and hippocampal volume among older men. Even though longitudinal research is necessary to elucidate the exact pathways of brokerage influence, our research has taken a step in the direction of examining the benefits of occupying a bridging position in a social network.

## Methods

### Participants and procedure

This study used data of the Korean Social Life, Health, and Aging Project (KSHAP). The KSHAP was conducted in Township K, a rural town in South Korea. Initially, 591 participants aged 60 or older and their spouses were administered a social network survey to collect a complete social network data^53^. Of the participants, 194 participants were assessed with neuropsychological tests, including measurements of episodic memory. Of these participants, those who did not meet the exclusion criteria (n = 125) were included in the analysis as neuropsychological test sample. Among these 125 participants who completed both the social network survey and neuropsychological tests, 65 participants (25 males and 40 females) were available for structural MRI data acquisition. Table 2 describes the characteristics of the participants. As shown in Table 2, demographic variables, depressive symptoms, memory function, and social network characteristics were assessed in the neuropsychological test sample. Subsequently, bilateral hippocampal volume and intracranial volume were measured in the 65 MRI subsample. In both the neuropsychological and the MRI subsamples, gender difference was not observed, except for years of education and intracranial volume. The study was approved by and administered in accordance with the relevant guidelines and regulations by the Institutional Review Board of Yonsei University, and all participants provided written informed consent for the research procedure.

**Table 2:** Demographic statistics.

	Neuropsychological test sample			MRI subsample		
	*n* = 125			*n* = 65		
	Women *n* = 74	Men *n* = 51		Women *n* = 40	Men *n* = 25	
	Mean (SD)	Mean (SD)	t	Mean (SD)	Mean (SD)	t
Age	71.45 (7.1)	72.57 (5.8)	−0.97	71.20 (6.7)	72.52 (6.02)	−0.82
Education	4.65 (3.36)	8.71 (3.59)	−6.37**	4.60 (2.9)	9.00 (3.65)	−5.14^**^
Depressive symptoms	12.03 (6.19)	11.75 (6.51)	0.24	12.00 (6.3)	11.84 (6.18)	0.10
**EVLT index**						
LTM Recall	1.66 (0.43)	1.54 (0.44)	1.53	1.69 (0.44)	1.64 (0.48)	0.42
LTM Recognition	2.35 (0.33)	2.35 (0.27)	−0.02	2.37 (0.29)	2.38 (0.26)	−0.16
**Social network characteristics**						
Brokerage	0.57 (0.29)	0.52 (0.31)	0.82	0.57 (0.29)	0.48 (0.31)	1.13
Social network size	2.24 (1.42)	2.67 (1.96)	−1.32	2.08 (1.23)	2.64 (1.93)	−1.31
**Brain volume**						
Left hippocampus volume	—	—		3574.57 (438.85)	3677.67 (435.88)	−0.93
Right hippocampus volume	—	—		3775.54 (454.29)	3823.036 (478.55)	−0.40
Intracranial Volume	—	—		1.23 (0.14)	1.39 (0.14)	4.60^**^

Education: years of formal education; Depressive Symptoms: Geriatric Depression Scale total score; EVLT index: Elderly Verbal Learning Test index; LTM: Long-Term Memory; Brokerage: Social network structural constraint (range 0–1), a smaller value indicates a larger opportunity for brokerage; Social Network Size: number of social ties in the global network; Intracranial Volume: Estimated total intracranial volume (/1,000,000 *mm*^3^). t = paired sample t-test result indicating a gender difference in variables. ^**^p < 0.001, 2-tailed.

**Table 3:** Linear regression models.

Neuropsychological test sample (n = 125)	All (n = 125)		Men (n = 51)		Women (n = 74)	
(a) Predicting Long-Term Memory Recognition	B (SE)	p	B (SE)	p	B (SE)	p
Constant	2.42 (0.3)	<0.01	2.55 (0.48)	<0.01	2.35 (0.39)	<0.01
Age	0 (0)	0.34	−0.01 (0.01)	0.28	0 (0.01)	0.70
Education	0.1 (0.02)	<0.01	0.1 (0.04)	0.01	0.1 (0.03)	<0.01
Depressive symptoms	−0.01 (0)	<0.01	−0.01 (0.01)	0.13	−0.02 (0.01)	0.00
Brokerage	0.07 (0.08)	0.41	0.01 (0.11)	0.93	0.12 (0.11)	0.29
Depressive symptoms × Brokerage	0.01 (0.01)	0.29	−0.04 (0.02)	0.04	0.05 (0.02)	0.01
Gender	−0.13 (0.06)	0.02				
Depressive symptoms × Brokerage × Gender	−0.08 (0.02)	<0.01				
**MRI subsample (n** = **65)**	**All (n** = **65)**		**Men (n** = **25)**		**Women (n** = **40)**	
**(b) Predicting adjusted left hippocampal volume**	**B (SE)**	**p**	**B (SE)**	**p**	**B (SE)**	**p**
Constant	3800.98 (456.25)	<0.01	3694.14 (635.57)	<0.01	3884.84 (635.57)	<0.01
Age	−14.05 (6.36)	0.03	−14.78 (8.89)	0.09	−13.65 (8.89)	0.13
Depressive symptoms	−2.12 (5.95)	0.72	−9.79 (9.42)	0.24	5.66 (9.42)	0.55
Brokerage	−149.41 (121.72)	0.22	−366.58 (172.18)	0.03	−9.77 (172.18)	0.96
Depressive symptoms × Brokerage	19.84 (18.57)	0.29	−42.30 (25.56)	0.10	58.50 (25.56)	0.03
Gender	−292.76 (74.22)	<0.01				
Depressive symptoms × Brokerage × Gender	−100.09 (38.23)	0.01				

B = Unstandardized coefficient, SE = Standard error.

Brokerage: Social network structural constraint (range 0–1), a smaller value indicates a larger opportunity for brokerage; Social network size: number of social ties in the global network.

### Exclusion criteria

Participants were excluded if they had any history of psychiatric or neurological disorders, vision or hearing problems, metals in their body that could not be removed, hypertension or diabetes uncontrollable with drugs or insulin, a history of losing consciousness due to head trauma, infarction, or a history of stroke. Participants who scored below the fifth percentile on the Long-Term Memory Recall Index in Elderly Memory Disorder Scale^54^ and below 1.5 SD on the Mini-Mental State Examination for Dementia Screening^55^ were interviewed based on the Clinical Dementia Rating Scale. They were then excluded from the analysis unless the interview revealed that their low cognitive performance was not due to preclinical symptoms of dementia. Among the participants who completed the entire imaging protocol, those who had either diffuse old infarction or excessive head motion that degraded the quality of an image were excluded. Furthermore, participants who had no or only one social tie were excluded because it was not possible to calculate a social network index indicating the opportunity for brokerage.

### Social network characteristics

Social network variables were created using data from the third wave of the KSHAP. The KSHAP adopted a name generator from the General Social Survey and National Social Life, Health, and Aging Project^53,56^. Individuals’ social connections were constructed from a name generator that identifies social network members, including a spouse, if any, and up to five discussion partners, with information on real names, gender, and residence. The questionnaire was as follows: “From time to time, most people discuss things that are important to them with others. For example, good or bad things that happen to you, problems you are having, or important concerns you may have. Looking back over the last 12 months, who are the people with whom you most often discussed things that were important to you?”.

To combine each respondent-centered network into a complete network of Township K, the people who appeared in different respondents’ networks were identified as identical based on the following criteria: (1) those who were not the spouses of respondents and lived outside of Township K were excluded, (2) at least two out of three Korean characters in their names matched, (3) their gender was the same, 4) their age difference was less than five years, and (5) their addresses belonged to the same Ri (the smallest administration unit in South Korea). Finally, we identified a complete network of 835 township residents and their 1,285 ties.

Social network constraint was used to measure the extent of bridging role (or brokerage). It is an inverse measure of brokerage since brokerage is supposed to reduce the network constraint^57,58^. The network constraint that person j exercises on person i comprises two parts: person i is constrained by person j as much as 1) person i invests time and resources into a relationship with person j (*p*_*ij*_), and 2) person i maintains a relationship with person k who has a connection with person j (*p*_*ik*_*p*_*kj*_). We used *p*_*ij*_ to represent the ratio of the social tie between i and j to the total ties of i and calculated it as follows: ${p}_{ij}=({a}_{ij}+{a}_{ji})/{\sum }_{k}({a}_{ik}+{a}_{ki})$, where *a*_*ij*_  =  1 means i responded that i has a tie with j. Otherwise, *a*_*ij*_  =  0. Thus, the formula to obtain an individual i's network constraint is calculated as ${c}_{i}=\sum _{j,\,j\ne i}\,{({p}_{ij}+\sum _{k,k\ne i,k\ne j}{p}_{ik}{p}_{kj})}^{2}$ in the following analysis. Social network size was measured by the number of social connections reported by each individual.

**Figure 4:**
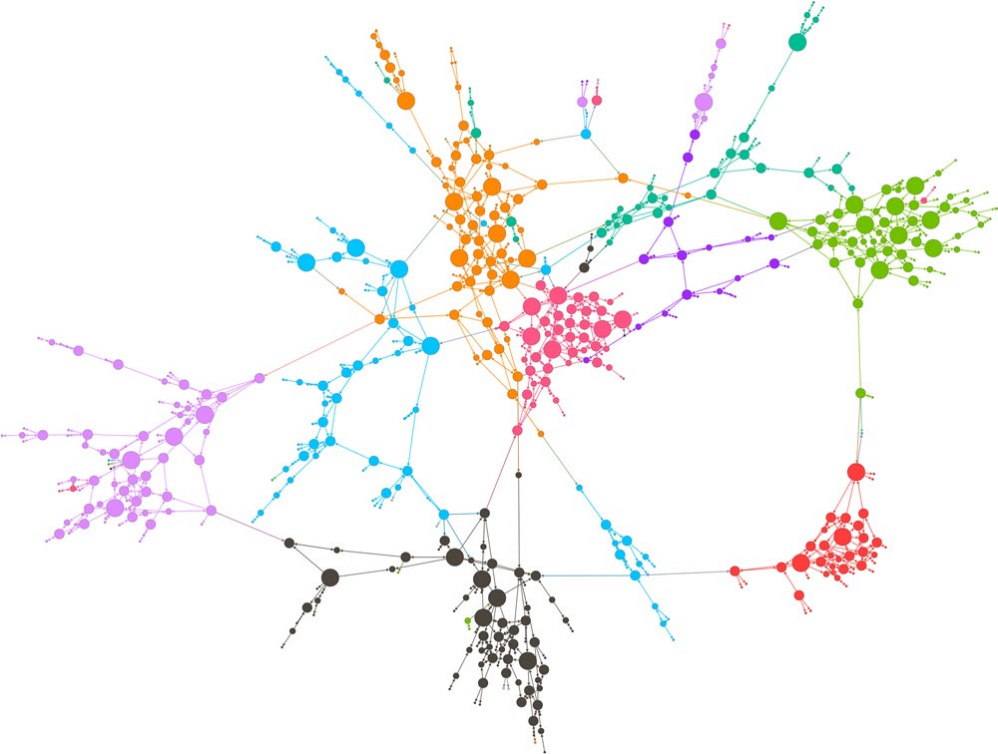
Complete social network that visualizes participants in the brokerage position. The size of the vertex indicates the amount of the individual’s opportunity for brokerage. The different colors represent different Ri.

Figure 1 describes more detailed characteristics of brokerage in social network. In the figure, A has ties with people who are connected to each other, and this type of social network is described as a highly constrained network: A is more likely to be constrained by the same strong demands from surrounding people because they are connected to each other and share the same norms and attitudes and exert influences on person A unanimously. In contrast with A, B maintains a relationship with others who are not connected to each other; thus, B occupies a brokerage position and enjoys fewer constraints but has to deal with diverse norms and demands from people with various backgrounds. Even though A and B have the same number of connections in their social network, presumably B has more brokerage potential and benefits from fewer constraints. Figure 4 offers a visualization of the complete social network in our study, indicating participants located in the brokerage position. Different colors indicate different Ri (the smallest administration unit in South Korea) in the village, and the size of each vertex represents the opportunity for bridging in their social network.

### Neuropsychological assessment

In the neuropsychological test sample, the Elderly Memory disorder Scale (EMS)^54^, a standardized neuropsychological battery, was administered to measure episodic memory. The EMS includes the Elderly Verbal Learning Test (EVLT), the Story Recall Test (SRT), and the Simplified Rey Figure Test (SRFT) as tests for episodic memory. EVLT is a nine-word, three-category verbal learning test utilizing the California Verbal Learning Test paradigm. SRT requires the memorization and retrieval of a paragraph of a story, containing 24 semantic units and 6 theme units. SRFT, which measures non-verbal memory, is a simplified version of the Rey–Osterreith Complex Figure for older adults. Based on each subset of episodic memory tests, the Long-Term Memory (LTM) Recall index was calculated by adding up the correct rate of Delayed Free Recall of the EVLT, the SRT, and the SRFT. The LTM Recognition index was calculated in an identical way using the correct rate of Delayed Recognition in EVLT, SRT, and SRFT.

### Assessment of depressive symptoms

Depressive symptoms were assessed using the Geriatric Depression Scale (GDS), a self-rating measure of depressive symptoms designed to screen late-life depression^59^. It includes 30 yes or no questions about an individual’s depressive mood. The GDS has high test-retest reliability (0.80 to 0.98)^59^, and it is widely used among community-dwelling older adults^60^. The GDS was translated into Korean by two psychiatrists and two clinical psychologists, but the original meaning of the items has been maintained^61^.

### Magnetic resonance imaging acquisition and regional volumetry

MRI images were acquired using a 3-Tesla MAGNETOM Trio 32 channel coil; 1 mm 224 sagittal slices of a whole brain T1-weighted image were acquired for each participant, with a MPRAGE sequence using the following parameters: TR = 2300 ms, TE = 2.3 ms, FOV = 256 × 256 mm, and FA = 9°. The time gap between social network measurement and MRI acquisition was 16 to 21 months. T1-weighted images were introduced into an automated segmentation procedure using FreeSurfer software 5.3 (http://surfer.nmr.mgh.harvard.edu/). This procedure has high accuracy comparable with manual labeling^62^. Bilateral hippocampal volume was proportionally adjusted with total intracranial volume (ICV)^63^.

### Statistical analysis

Statistical analysis was conducted using the SPSS package (version 22; IBM, USA). Preliminarily, Pearson correlation analysis was performed not only to describe the associations among the variables of interest, age, and years of education but also to determine whether they should be registered as covariates in the following analysis. In addition, an independent t-test was conducted to investigate possible gender differences in the study variables.

We used PROCESS macro 2.16 implemented in SPSS^64^ to generate and test multiple regression models predicting LTM indices and bilateral hippocampal volume using the neuropsychological test sample and the MRI subsample population, respectively. First, we examined whether the effect of depressive symptoms (*X*) on the LTM indices (*Y*) is moderated by brokerage (*M*) among men and women. Then, we examined whether this moderation effect (*XM*) is additionally moderated by gender (*W*). Therefore, gender-specific multiple regression models included the two-way interaction term (*XM*), and the following model included the three-way interaction term (*XMW*). Both models were tested in the neuropsychological test sample. Age and education were adjusted as covariates of no interest. Subsequent to this analysis, to predict bilateral hippocampal volume, an identical statistical procedure was repeated in the subsampled population who had undergone an MRI scan, adjusting for the effect of age.

## Data Availability

The datasets generated during and/or analyzed during the current study are available in the Open Science Framework repository at https://osf.io/56cfm/.
